# Indoxyl Sulfate Promotes Vascular Calcification in Association with Oxidative Stress and Activation of ERK and Wnt/β-Catenin Signaling Pathways

**DOI:** 10.3390/ijms27167314

**Published:** 2026-08-16

**Authors:** Yi-Cheng Wang, I-Min Su, Chung-Jen Lee, Tsung-Jui Wu, Bang-Gee Hsu

**Affiliations:** 1 Division of Nephrology, Hualien Tzu Chi Hospital, Buddhist Tzu Chi Medical Foundation, Hualien 97004, Taiwan; 2 Department of Anesthesiology, Dalin Tzu Chi Hospital, Buddhist Tzu Chi Medical Foundation, Chiayi 62247, Taiwan; 3 School of Medicine, Tzu Chi University, Hualien 97004, Taiwan; 4 Institute of Medical Sciences, Tzu Chi University, Hualien 97004, Taiwan; 5 Department of Nursing, Tzu Chi University, Hualien 97004, Taiwan; 6 Division of Nephrology, Department of Medicine, Hualien Armed Forces General Hospital, National Defense Medical Center, Hualien 97144, Taiwan

**Keywords:** reactive oxygen species, chronic kidney disease, vascular smooth muscle cells, indoxyl sulfate, vascular calcification

## Abstract

Vascular calcification (VC) is a major complication of chronic kidney disease (CKD) that is strongly associated with cardiovascular mortality. While indoxyl sulfate (IS), a protein-bound uremic toxin, has been implicated in the progression of VC, the underlying molecular mechanisms remain unclear. We investigated the procalcific effects of IS using a two-step nephrectomy-induced CKD mouse model and cultured vascular smooth muscle cells. In vivo, progressive renal impairment was associated with elevated circulating IS levels and enhanced VC. In vitro, IS dose- and time-dependently induced calcium deposition, increased reactive oxygen species (ROS) production, and upregulated osteogenic markers, including runt-related transcription factor 2 (RUNX2), bone morphogenetic protein 2 (BMP2), and osteocalcin (OCN), whereas N-acetyl-L-cysteine (NAC) partially attenuated IS-induced ROS accumulation and cell injury. Mechanistically, IS exposure activated extracellular signal-regulated kinase (ERK) and Wnt/β-catenin signaling while suppressing nuclear factor erythroid 2-related factor 2 (Nrf2)-related antioxidant responses, as reflected by reductions in the phosphorylated Nrf2 (pNrf2) to total Nrf2 and heme oxygenase-1 (HO-1) expression. Pharmacological inhibition of ERK and Wnt/β-catenin signaling attenuated IS-induced osteogenic responses. These findings indicate that IS promotes VC in association with increased oxidative stress, activation of ERK and Wnt/β-catenin signaling, and impaired Nrf2-related antioxidant defense. These integrated findings provide mechanistic insight into IS-associated VC and highlight oxidative stress-related signaling networks as potential therapeutic targets in CKD.

## 1. Introduction

Chronic kidney disease (CKD) is a major global health burden and is strongly associated with cardiovascular complications, particularly vascular calcification (VC), which is a key predictor of cardiovascular morbidity and mortality [1,2]. Renal fibrosis represents a common pathological pathway in progressive CKD and is characterized by excessive extracellular matrix accumulation, persistent inflammation, oxidative stress, tubular epithelial injury, endothelial dysfunction, and maladaptive tissue repair [3,4,5]. At the molecular level, CKD progression and renal fibrosis are regulated by multiple interconnected pathways, including transforming growth factor-β/Smad, nuclear factor-κB, renin–angiotensin system, aryl hydrocarbon receptor (AhR), nuclear factor erythroid 2-related factor 2 (Nrf2), and Wnt/β-catenin signaling [5,6,7]. These pathways contribute not only to progressive renal structural remodeling but also to a systemic proinflammatory and prooxidative milieu that may promote vascular injury and calcification. Accumulation of uremic toxins is another hallmark of CKD progression, and protein-bound solutes, such as indoxyl sulfate (IS), have been increasingly recognized as important mediators linking renal dysfunction to vascular injury [8,9]. Derived from gut microbiota metabolism of dietary tryptophan, IS is poorly cleared by conventional dialysis due to its high protein-binding affinity [10]. Emerging evidence indicates that IS promotes osteogenic differentiation of vascular smooth muscle cells (VSMCs), thereby promoting medial calcification [11,12]. However, the precise molecular mechanisms underlying IS-induced VC, particularly the integration of oxidative stress-related signaling pathways, have only been partially elucidated.

Oxidative stress is a central driver of vascular calcification, particularly by inducing phenotypic switching of VSMCs toward an osteogenic state. Excessive reactive oxygen species (ROS) production activates multiple downstream signaling pathways, including mitogen-activated protein kinase (MAPK) cascades, leading to the upregulation of osteogenic transcription factors, such as Runt-related transcription factor 2 (RUNX2) [13,14,15]. Simultaneously, oxidative stress disrupts cellular antioxidant defenses, notably by impairing Nrf2 signaling, further exacerbating redox imbalance and vascular injury [16,17]. Since IS induces ROS generation and promotes osteogenic transformation in VSMCs [18], oxidative stress is likely an upstream driver of multiple procalcific signaling pathways in IS-induced VC. However, the integration of these signaling events remains poorly understood.

The Wnt/β-catenin signaling pathway is one such pro-calcific pathway that critically regulates osteogenic differentiation in VSMCs. Activation of β-catenin signaling promotes the expression of osteogenic genes and contributes to VC [19,20]. Recent evidence suggests that oxidative stress may interact with Wnt/β-catenin and MAPK/extracellular signal-regulated kinase (ERK) signaling pathways, indicating potential crosstalk among these procalcific mechanisms [21,22,23]. However, the coordinated regulation of ERK and β-catenin signaling in response to IS-induced ROS generation remains unclear.

In this study, we investigated the procalcific effects of IS using a nephrectomy-induced CKD mouse model and cultured VSMCs. Specifically, we investigated the role of oxidative stress in mediating IS-induced VC to characterize the involvement of the ERK and Wnt/β-catenin signaling pathways. By integrating in vivo and in vitro findings, this study elucidated the molecular interplay among ROS generation, osteogenic signaling, and impaired antioxidant defense. These findings may provide a mechanistic basis for targeting oxidative stress-driven signaling networks and identifying potential therapeutic strategies to mitigate VC in CKD.

## 2. Results

### 2.1. Progressive Renal Dysfunction and Indoxyl Sulfate Accumulation in a Two-Step Nephrectomy-Induced Chronic Kidney Disease Mouse Model

**Figure 1:**
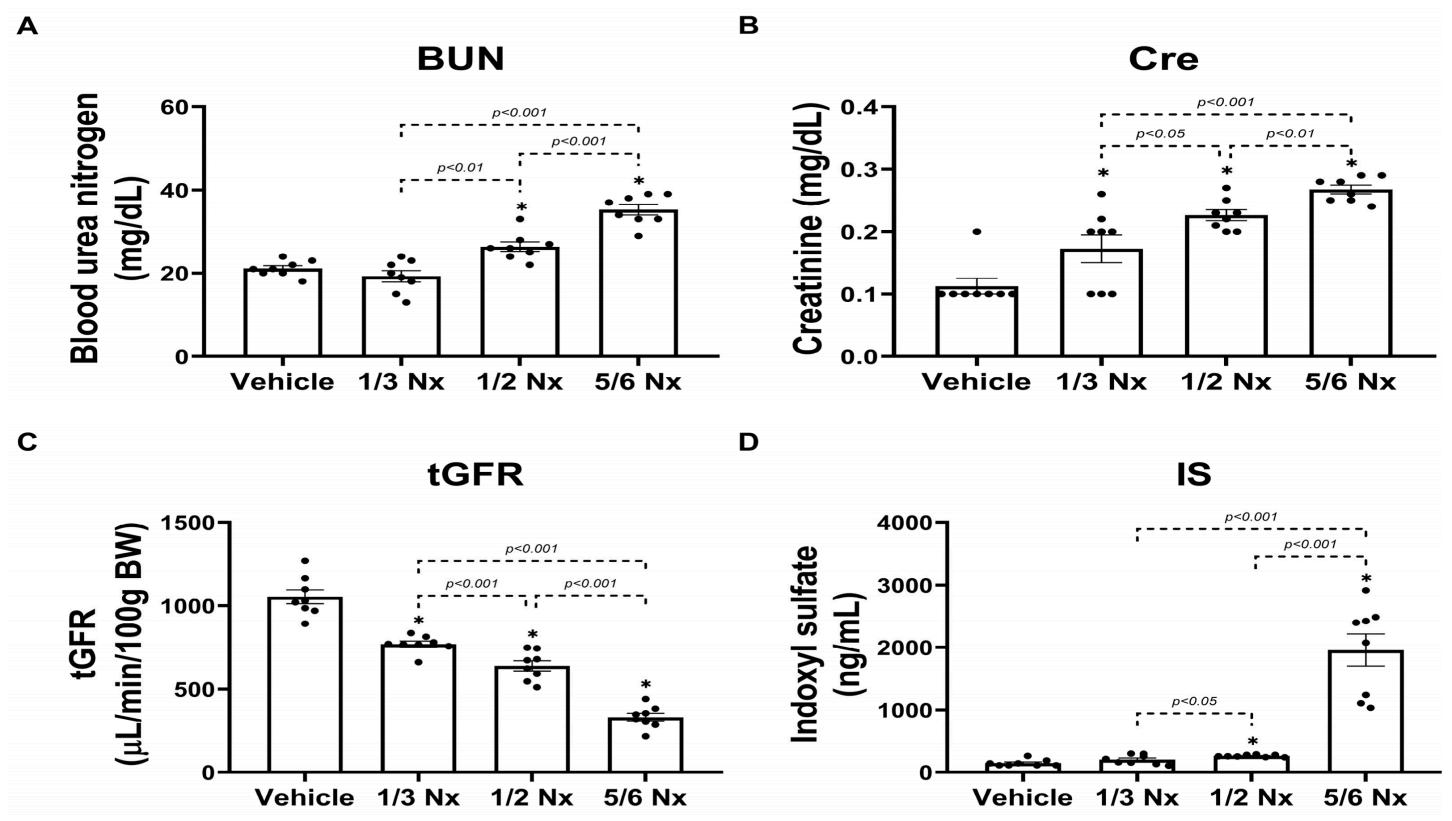
Renal function parameters and serum indoxyl sulfate levels in a two-step nephrectomy-induced chronic kidney disease mouse model. C57BL/6 mice were assigned to the Vehicle, 1/3 nephrectomy (1/3 Nx), 1/2 nephrectomy (1/2 Nx), and 5/6 nephrectomy (5/6 Nx) groups to generate different degrees of renal mass reduction. Serum levels of (**A**) blood urea nitrogen (BUN), (**B**) creatinine, (**C**) transdermal glomerular filtration rate (tGFR), and (**D**) serum indoxyl sulfate (IS) were assessed at 16 weeks of age. Progressive nephrectomy-induced renal dysfunction was accompanied by a marked increase in circulating IS levels. Each dot represents an individual mouse. Data are presented as mean ± standard error of the mean (*n* = 8 per group). Statistical analysis was performed using one-way analysis of variance followed by Tukey’s multiple-comparisons test. * *p* < 0.05 vs. Vehicle; *p* values for comparisons among nephrectomy groups are shown above the brackets.

Compared with the Vehicle group, serum blood urea nitrogen (BUN) levels were significantly increased in the 1/2 nephrectomy (1/2 Nx) and 5/6 nephrectomy (5/6 Nx) groups, whereas serum creatinine levels were significantly elevated in all nephrectomy groups (* *p* < 0.05; Figure 1A,B). In contrast, the transdermal glomerular filtration rate (tGFR) was significantly reduced in the 1/3 Nx, 1/2 Nx, and 5/6 Nx groups, with the greatest reduction observed in the 5/6 Nx group (* *p* < 0.05; Figure 1C). Serum IS levels were significantly elevated in nephrectomized mice and increased markedly with worsening renal impairment (* *p* < 0.05; Figure 1D).

### 2.2. Nephrectomy Severity Aggravated Aortic Calcium Accumulation on Von Kossa Staining

**Figure 2:**
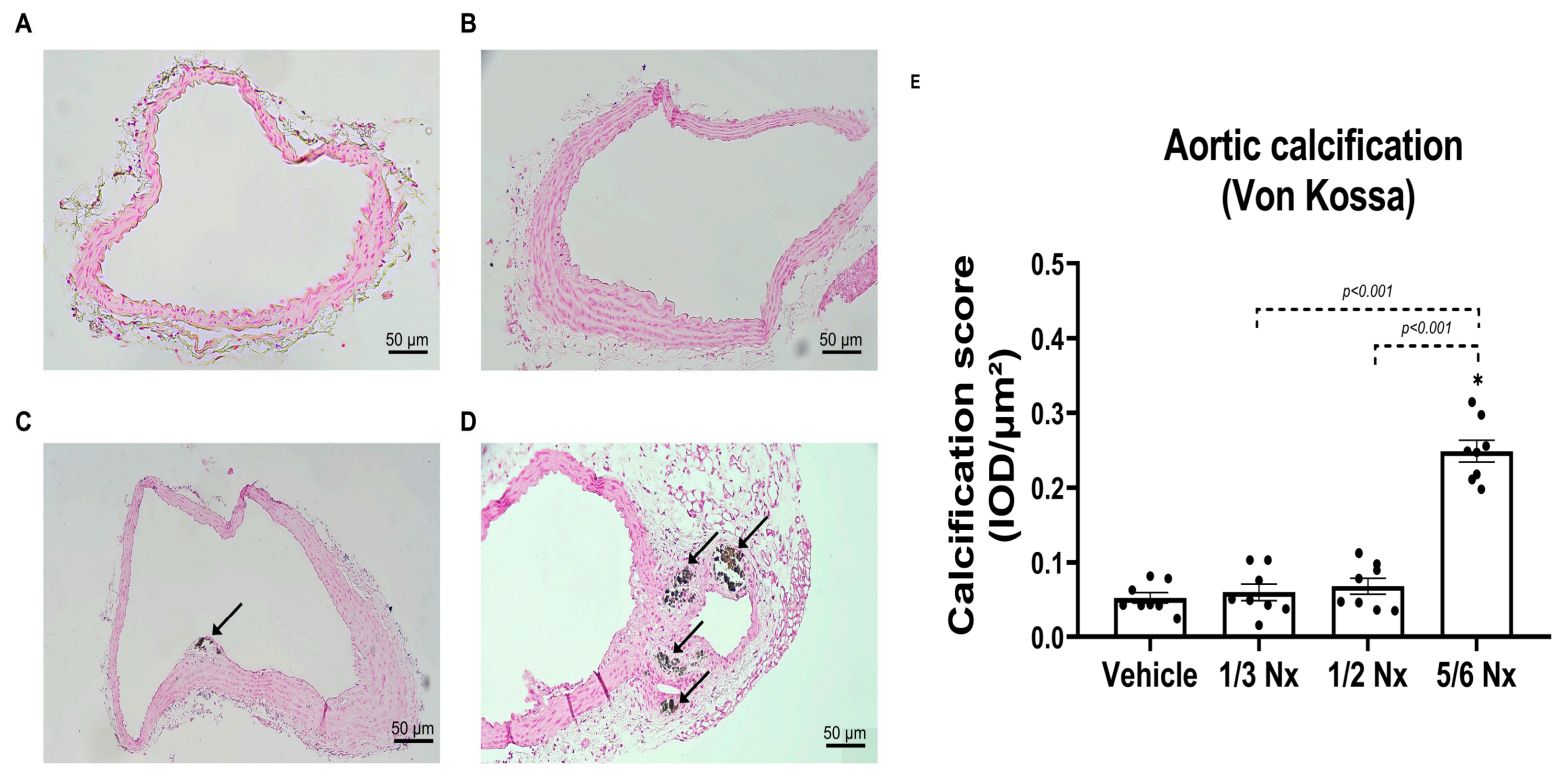
Nephrectomy severity aggravated aortic medial calcification in mice. Representative von Kossa-stained aortic sections from C57BL/6 mice in the (**A**) Vehicle, (**B**) 1/3 nephrectomy (1/3 Nx), (**C**) 1/2 nephrectomy (1/2 Nx), and (**D**) 5/6 nephrectomy (5/6 Nx) groups are shown. Black staining indicates calcium phosphate deposition within the aortic wall. Black arrows indicate representative areas of von Kossa-positive calcium phosphate deposition. (**E**) Quantitative analysis of vascular calcification was performed by measuring the calcified area and expressing it as integrated optical density per area (IOD/μm^2^). Aortic calcification increased progressively with the severity of renal mass reduction. Each dot represents an individual mouse. Data are presented as mean ± standard error of the mean (*n* = 8 mice per group). Statistical analysis was performed using one-way analysis of variance followed by Tukey’s multiple-comparisons test. * p < 0.05 vs. Vehicle; *p* values for comparisons among nephrectomy groups are shown above the brackets. Scale bars = 50 μm.

Von Kossa staining revealed progressive calcium deposition in the aortic medial layer of nephrectomized mice (Figure 2A–D), with increasing vascular calcification proportional to the extent of renal mass reduction. In contrast, minimal calcification was observed in the vehicle group. Von Kossa staining showed progressively greater calcium deposition as nephrectomy severity increased. Quantitative analysis demonstrated that aortic calcification was significantly increased in the 5/6 Nx group compared with the Vehicle group, whereas the differences in the 1/3 Nx and 1/2 Nx groups did not reach statistical significance (* p < 0.05; Figure 2E).

### 2.3. Indoxyl Sulfate-Induced Calcification in Mouse Aortic Vascular Smooth Muscle Cells in a Dose- and Time-Dependent Manner

**Figure 3:**
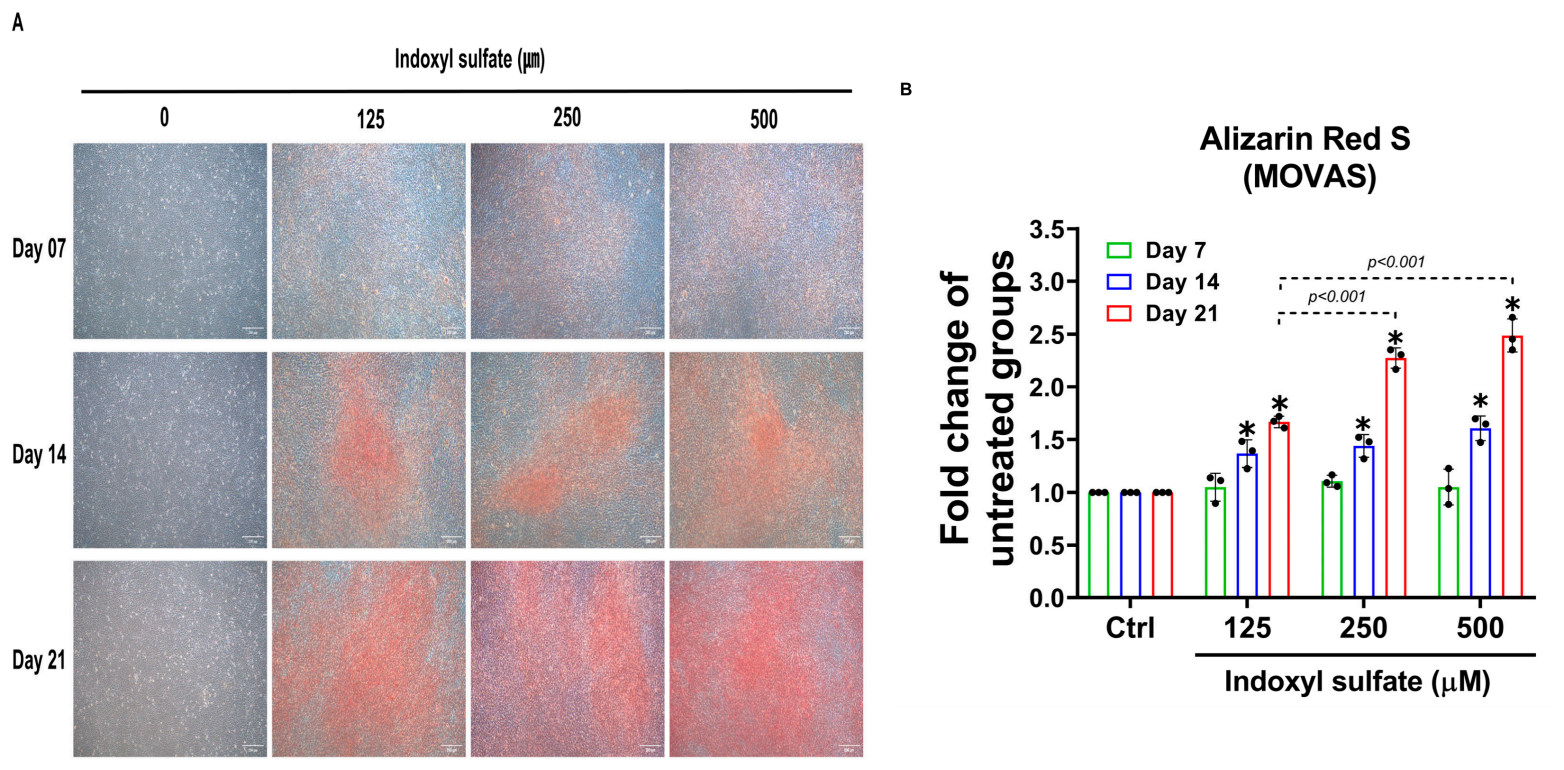
Indoxyl sulfate induced calcification in mouse aortic vascular smooth muscle cells in a dose- and time-dependent manner. Mouse aortic vascular smooth muscle cells (MOVAS cells) were treated with increasing concentrations of indoxyl sulfate (IS; 0, 125, 250, and 500 μM) for 7, 14, and 21 days. (**A**) Representative Alizarin Red S staining images show calcium deposition in MOVAS cells after IS exposure. Increased red staining indicates enhanced extracellular calcium deposition. (**B**) Quantification of Alizarin Red S staining was performed by dye extraction and absorbance measurement, and results are expressed as fold changes relative to the untreated control at the corresponding time point. IS treatment promoted calcium deposition in a concentration- and time-dependent manner. Data are presented as mean ± standard deviation (*n* = 3 independent experiments). Statistical analysis was performed using two-way analysis of variance followed by Šídák’s multiple-comparisons test. * p < 0.05 vs. control; *p* values for comparisons among IS concentrations at the same time point are shown above the brackets. Scale bars = 200 μM.

Alizarin Red S staining revealed increased calcium deposition in mouse aortic VSMCs (MOVAS) cells following IS treatment (Figure 3A). While minimal staining was observed in control cells, IS-treated cells exhibited a concentration- and time-dependent increase in calcification, with significantly pronounced effects at days 14 and 21. Quantitative analysis confirmed a significant increase in calcium deposition relative to untreated controls (* p < 0.05; Figure 3B), with the greatest effect observed at 500 µM IS.

### 2.4. Effects of N-Acetyl-L-Cysteine on Indoxyl Sulfate-Induced Oxidative Stress and Cell Viability in Mouse Aortic Vascular Smooth Muscle Cells

**Figure 4:**
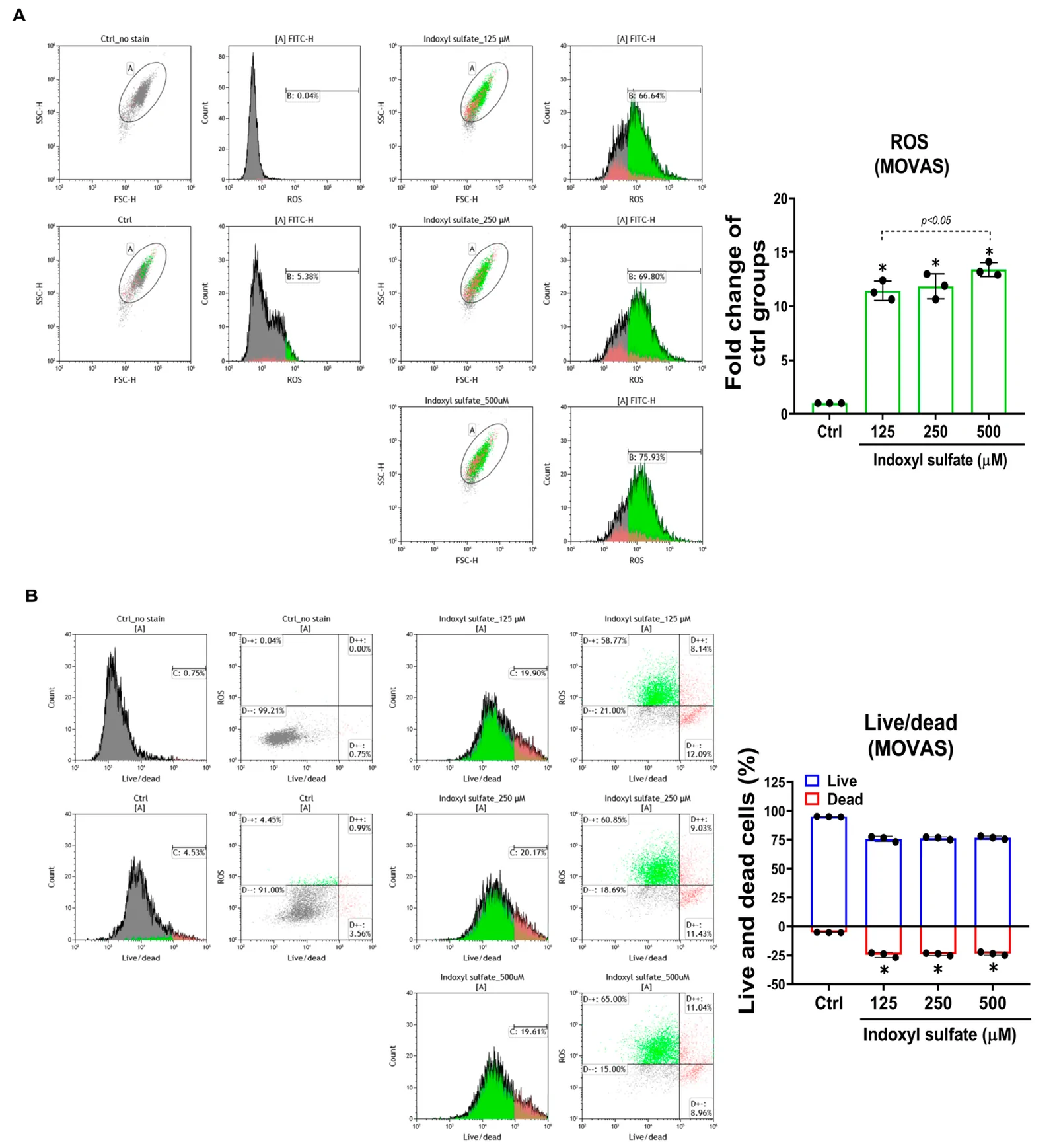

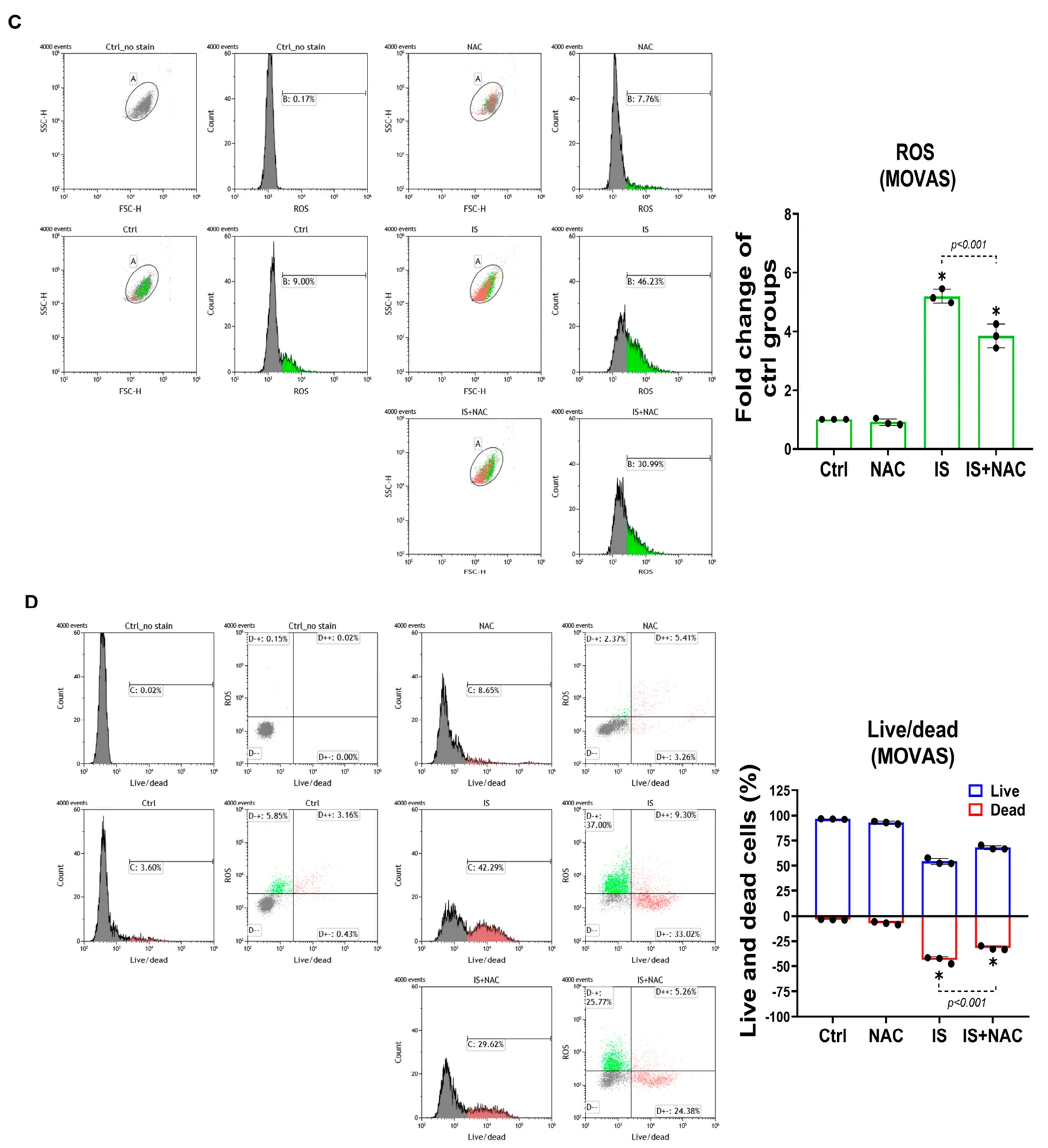
Indoxyl sulfate increased intracellular reactive oxygen species accumulation and reduced cell viability, whereas N-acetyl-L-cysteine attenuated these effects in mouse aortic vascular smooth muscle (MOVAS) cells. For the concentration-response experiments, MOVAS cells were treated with indoxyl sulfate (IS) at 0, 125, 250, or 500 μM for 21 days. (**A**) Intracellular reactive oxygen species (ROS) levels were assessed using 2′,7′-dichlorodihydrofluorescein diacetate (DCFDA) staining followed by flow cytometry. (**B**) Cell viability was assessed using a live/dead viability dye and flow cytometry. For the antioxidant experiments, MOVAS cells were divided into four groups: untreated control, N-acetyl-L-cysteine (NAC) alone, IS alone, and IS plus NAC. Cells were pretreated with 2 mM NAC for 1 h before exposure to 500 μM IS for 21 days. (**C**) Intracellular ROS levels were assessed using DCFDA staining and flow cytometry. (**D**) Cell viability was assessed using a live/dead viability dye and flow cytometry. Representative gating plots, fluorescence histograms, and bivariate flow cytometric plots are shown. ROS levels are expressed as fold changes relative to the untreated control, and cell viability results are expressed as the proportions of viable and nonviable cells. Dead-cell percentages were plotted below zero solely for graphical presentation. The horizontal brackets indicate the specified comparisons. Data are presented as mean ± standard deviation (*n* = 3 independent experiments). Gray, green, and red events represent viable ROS-negative, viable ROS-positive, and nonviable cells, respectively. Blue and red bars in the summary Live/Dead graphs represent live and dead cells, respectively. Statistical analysis was performed using one-way analysis of variance followed by Tukey’s multiple-comparisons test or Dunnett’s multiple-comparison test. In (**A**,**B**), asterisks indicate comparisons with the untreated control group. In (**C**,**D**), horizontal brackets indicate comparisons between the IS plus NAC group and the IS-alone group. * p < 0.05 vs. control; *p* values for comparisons among control and IS concentrations or between the IS and IS+NAC groups at the same time point are shown above the brackets.

Flow cytometric analysis showed that IS treatment increased intracellular ROS accumulation and reduced cell viability across the tested concentrations (Figure 4A,B). To further examine the contribution of oxidative stress, MOVAS cells were treated with N-acetyl-L-cysteine (NAC). NAC alone had little effect on basal ROS levels, whereas NAC co-treatment significantly attenuated the IS-induced increase in ROS production (Figure 4C). Consistently, NAC co-treatment partially preserved cell viability and reduced IS-induced cell death (Figure 4D). These findings support the contribution of oxidative stress to IS-induced cellular injury in MOVAS cells.

### 2.5. Indoxyl Sulfate-Induced Osteogenic Differentiation and Modulated Calcification-Related Signaling Pathways in Mouse Aortic Vascular Smooth Muscle Cells

**Figure 5:**
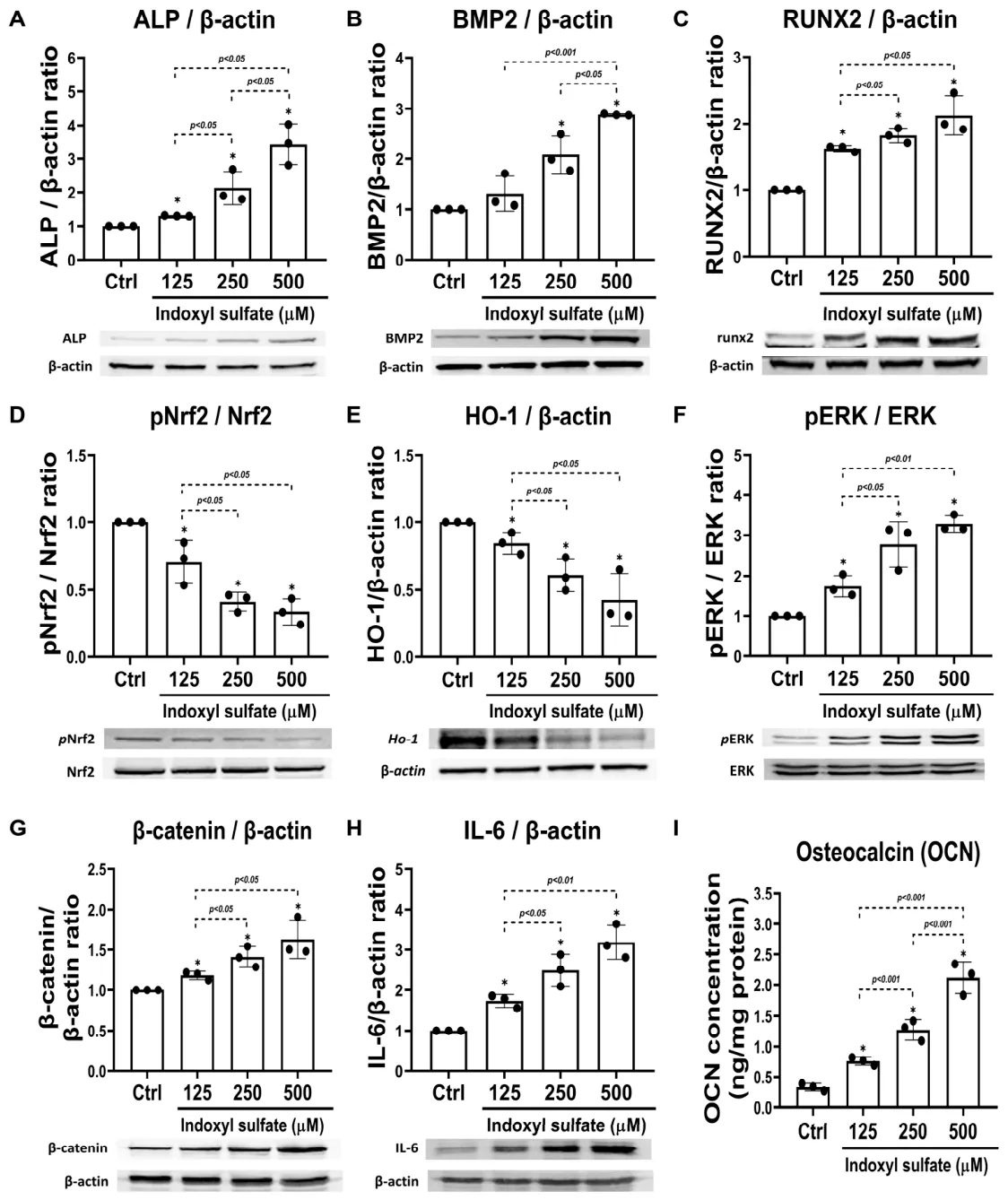
Indoxyl sulfate promoted osteogenic differentiation and modulated oxidative stress-related signaling pathways in mouse aortic vascular smooth muscle (MOVAS) cells. MOVAS cells were treated with indoxyl sulfate (IS) at 0, 125, 250, or 500 μM for 21 days. Western blotting and enzyme-linked immunosorbent assay (ELISA) were performed to assess osteogenic markers, antioxidant signaling proteins, inflammatory mediators, and procalcific signaling proteins. (**A**) alkaline phosphatase (ALP) expression normalized to β-actin. (**B**) bone morphogenetic protein 2 (BMP2) expression normalized to β-actin. (**C**) runt-related transcription factor 2 (RUNX2) expression normalized to β-actin. (**D**) the ratio of phosphorylated nuclear factor erythroid 2-related factor 2 to total Nrf2 (pNrf2/Nrf2). (**E**) heme oxygenase-1 (HO-1) expression normalized to β-actin. (**F**) the ratio of phosphorylated extracellular signal-regulated kinase to total ERK (pERK/ERK). (**G**) β-catenin expression normalized to β-actin. (**H**) interleukin-6 (IL-6) expression normalized to β-actin. (**I**) intracellular osteocalcin (OCN) concentrations measured by ELISA and normalized to total cellular protein content, expressed as ng/mg protein. Representative immunoblots are shown below the corresponding quantitative panels in (**A**–**H**). Data are presented as the mean ± standard deviation from three independent experiments. Statistical analysis was performed using one-way analysis of variance followed by Tukey’s multiple-comparisons test, with the untreated control group as the reference group. Horizontal brackets indicate comparisons between the specified IS-treated groups and the untreated control group. * *p* < 0.05 vs. control; exact *p* values for comparisons among IS-treated groups are shown above the brackets.

Western blot analysis revealed that IS promoted osteogenic differentiation in MOVAS cells, as evidenced by increased expression of alkaline phosphatase (ALP), bone morphogenetic protein 2 (BMP2), and RUNX2 (Figure 5A–C). Consistently, intracellular osteocalcin (OCN) concentrations measured by enzyme-linked immunosorbent assay (ELISA) were significantly increased following IS treatment (Figure 5I), further supporting IS-induced osteogenic differentiation. To evaluate antioxidant and procalcific signaling, we subsequently examined Nrf2-related and downstream signaling proteins. IS treatment reduced the pNrf2/Nrf2 ratio and heme oxygenase-1 (HO-1) expression (Figure 5D,E), indicating impaired antioxidant responses, while increasing the pERK/ERK ratio and β-catenin expression (Figure 5F,G). Interleukin-6 (IL-6) expression was also increased following IS exposure (Figure 5H).

### 2.6. Inhibition of Wnt/β-Catenin and Extracellular Signal-Regulated Kinase Signaling Attenuated Indoxyl Sulfate-Induced Osteogenic Responses in Mouse Aortic Vascular Smooth Muscle Cells

**Figure 6:**
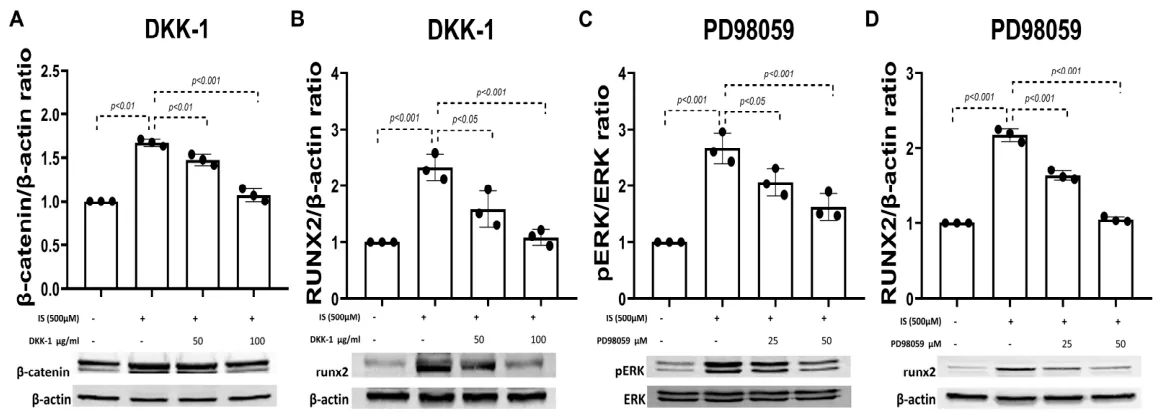
Pharmacological inhibition of Wnt/β-catenin and extracellular signal-regulated kinase (ERK) signaling attenuated indoxyl sulfate-induced osteogenic responses in mouse aortic vascular smooth muscle (MOVAS) cells. MOVAS cells were initially exposed to indoxyl sulfate (IS; 500 μM), followed by treatment with either the Wnt/β-catenin pathway inhibitor recombinant Dickkopf-1 (DKK-1; 50 or 100 μg/mL) or the ERK pathway inhibitor PD98059 (25 or 50 μM). Protein lysates were collected 2 h after the addition of DKK-1 or PD98059 and subjected to Western blot analysis. (**A**) β-catenin expression normalized to β-actin following treatment with DKK-1. (**B**) runt-related transcription factor 2 (RUNX2) expression normalized to β-actin following treatment with DKK-1. (**C**) the ratio of phosphorylated extracellular signal-regulated kinase to total ERK (pERK/ERK) following treatment with PD98059. (**D**) RUNX2 expression normalized to β-actin following treatment with PD98059. Representative immunoblots are shown below the corresponding quantitative panels. Data are presented as the mean ± standard deviation from three independent experiments. Statistical analysis was performed using one-way analysis of variance followed by Dunnett’s multiple-comparisons test, with the IS-alone group as the reference group. The *p*-values for comparisons with the IS-alone group are shown above the brackets.

The involvement of the Wnt/β-catenin and ERK signaling pathways was further validated through pharmacological interventions. Treatment with the Wnt pathway inhibitor Dickkopf-like protein (DKK-1) reduced β-catenin expression in IS-treated MOVAS cells (Figure 6A), with an accompanying decrease in RUNX2 expression (Figure 6B), implicating β-catenin signaling in osteogenic differentiation. Similarly, inhibition of ERK signaling through PD98059 treatment decreased the pERK/ERK ratio (Figure 6C) and reduced RUNX2 expression (Figure 6D), indicating that ERK activation contributes to IS-induced osteogenic responses.

## 3. Discussion

In this study, we found that IS promoted calcific and osteogenic responses in MOVAS cells, as demonstrated by increased calcium deposition and elevated ALP, RUNX2, BMP2, and OCN levels. IS exposure was also associated with enhanced ROS generation, ERK phosphorylation, β-catenin upregulation, increased IL-6 expression, and suppression of Nrf2-related antioxidant responses, as reflected by reductions in the pNrf2/Nrf2 ratio and HO-1 expression. NAC partially attenuated IS-induced ROS accumulation and loss of cell viability, providing functional evidence that oxidative stress contributes to IS-induced cellular injury. In addition, pharmacological inhibition of ERK and Wnt/β-catenin signaling reduced IS-induced osteogenic responses, supporting the functional involvement of these pathways in the osteogenic effects of IS.

Previous studies have established IS as a key uremic toxin that contributes to VC primarily by inducing oxidative stress and promoting osteogenic differentiation in VSMCs [24]. Consistent with these findings, our study demonstrates that IS exposure increased ROS production and upregulated multiple osteogenic markers, including RUNX2, BMP2, and OCN, supporting osteogenic and calcific responses in MOVAS cells. Nevertheless, the phenotypic characterization in the present study remains incomplete. Although increases in ALP, RUNX2, BMP2, and OCN support activation of an osteogenic program, we did not assess PiT-1 or Osterix, nor did we evaluate the loss of contractile VSMC markers such as SM22α. Therefore, the present findings should be interpreted as evidence of osteogenic and calcific responses rather than as a comprehensive demonstration of the full contractile-to-osteogenic phenotypic transition. Earlier studies have also implicated MAPK signaling pathways, including ERK, in the mediation of oxidative stress-induced VC [25]. Our findings are likewise consistent with these observations and further support the role of ERK signaling in IS-induced osteogenic responses. Notably, our study expands on current knowledge by demonstrating the coordinated regulation of multiple procalcific signaling pathways downstream of oxidative stress. While previous studies have primarily focused on individual pathways, such as ERK activation, our findings verify the concurrent activation of Wnt/β-catenin signaling in response to IS exposure. Thus, our findings suggest that ROS may act as an upstream mediator associated with ERK and β-catenin activation while concurrently impairing Nrf2-dependent antioxidant defenses. The relationship between IS, oxidative stress, and inflammation should also be interpreted in the context of the gut–kidney axis. IS is a representative gut-derived protein-bound uremic toxin generated from dietary tryptophan metabolism by intestinal microbiota [26]. During CKD progression, impaired renal clearance and gut dysbiosis promote the accumulation of IS and other microbiota-derived metabolites, thereby contributing to a systemic prooxidative and proinflammatory environment [26,27]. Recent studies have emphasized that the microbiota–gut–kidney axis plays an important role in renal disease progression, renal fibrosis, and the accumulation of uremic toxins [26,27]. Consistently, clinical data have shown that total and free IS levels are negatively correlated with estimated glomerular filtration rate across CKD stages, supporting the close relationship between IS accumulation and renal functional decline [28]. Mechanistically, IS has been linked to aryl hydrocarbon receptor-dependent signaling, ROS generation, nuclear factor-κB activation, and inflammatory cytokine production [29]. In the present study, IS exposure increased ROS levels and IL-6 expression while reducing the pNrf2/Nrf2 ratio, suggesting that IS induces an imbalance between oxidative stress generation and antioxidant defense. These findings support the concept that IS-mediated VC is not driven by osteogenic signaling alone but is closely associated with oxidative and inflammatory pathway activation. Beyond operating as a simple upstream trigger, ROS may function as a central regulator that coordinates multiple procalcific pathways [30]. Oxidative stress in VSMCs drives phenotypic switching from a contractile to an osteogenic state, a key process underlying VC [31]. Recent studies further support the role of ROS in promoting osteogenic or osteochondrogenic differentiation of VSMCs. Hypoxia has been shown to induce osteochondrogenic differentiation and extracellular matrix calcification of VSMCs through hypoxia-inducible factor-1 and ROS-dependent mechanisms [32]. In addition, sirtuin 7 was recently reported to protect against VC by regulating intracellular ROS accumulation and VSMC senescence, and ROS inhibition attenuated VSMC calcification in that model [33]. In this scenario, the simultaneous activation of ERK and β-catenin signaling, together with the suppression of Nrf2-dependent antioxidant defense, may create a self-amplifying procalcific environment [34,35]. Increased ROS activates osteogenic signaling pathways and impairs cellular antioxidant capacity, further promoting redox imbalance and sustaining osteogenic transformation. The suppression of Nrf2 signaling reinforces the role of oxidative stress in IS-induced VC. Nrf2 is a key regulator of cellular antioxidant defense, and its impairment has been associated with enhanced VC and redox imbalance [36,37]. In our study, suppression of the Nrf2-related antioxidant response was reflected by reductions in both the pNrf2/Nrf2 ratio and HO-1 expression, which may exacerbate ROS accumulation and facilitate activation of downstream procalcific pathways. This dual effect of increased ROS generation and impaired antioxidant defense implies a critical mechanism underlying IS-induced vascular injury.

Pharmacological inhibition revealed functional evidence supporting the involvement of ERK and Wnt/β-catenin signaling pathways in IS-induced osteogenic differentiation. The observed activation of Wnt/β-catenin signaling by IS is biologically plausible and is consistent with emerging evidence linking gut microbiota-derived tryptophan metabolites, AhR signaling, and Wnt/β-catenin pathway activation in CKD-related renal fibrosis [38]. Recent studies have suggested that microbial-mediated tryptophan metabolism can promote renal fibrosis through AhR-dependent hyperactivation of Wnt/β-catenin signaling. Although these findings were primarily derived from renal fibrosis models, they support a broader mechanistic link among gut-derived uremic toxins, AhR-related signaling, and Wnt/β-catenin activation in CKD. In VSMCs, Wnt/β-catenin signaling is a well-recognized procalcific pathway that promotes osteogenic transdifferentiation and RUNX2 expression [19,20]. In the present study, IS increased β-catenin and RUNX2 expression, whereas DKK-1 reduced IS-induced β-catenin and RUNX2 expression. These findings suggest that Wnt/β-catenin signaling contributes functionally to IS-induced osteogenic responses and may represent a downstream pathway linking the accumulation of gut-derived uremic toxins to VC in CKD. Inhibition of β-catenin signaling by DKK-1 and ERK signaling by PD98059 attenuated RUNX2 expression, indicating the involvement of both pathways in the osteogenic response. However, these pathways may act in parallel or exhibit crosstalk, necessitating further studies to clarify their hierarchical relationship and their upstream regulatory mechanisms. The in vivo and in vitro findings of this study collectively provide a comprehensive view of IS-induced VC. The nephrectomy-induced CKD model demonstrated progressive accumulation of IS and increased VC, while in vitro experiments established the direct effects of IS on VSMCs. These complementary approaches strengthen the biological relevance of our findings and support the translational significance of IS-mediated oxidative stress in CKD-associated VC. The clinical relevance of IS accumulation has also been supported by human studies. In patients with CKD, higher serum IS levels have been associated with VC, arterial stiffness, and adverse cardiovascular outcomes [39]. A clinical cohort study reported that serum IS was associated with vascular disease, VC, and mortality in CKD patients [39]. In addition, serum IS levels have been shown to predict major adverse cardiovascular events in patients with CKD, supporting its potential role as a cardiovascular risk marker [40]. Recent clinical evidence also indicates that total and free IS concentrations increase across categories of declining glomerular filtration rate and are inversely correlated with estimated glomerular filtration rate, further linking IS accumulation to renal functional decline [28]. Together with recent reviews emphasizing uremic toxin-driven vascular inflammation and calcification in CKD [41,42], these clinical studies support the translational significance of our experimental findings. However, because most clinical evidence remains observational, further longitudinal and interventional studies are required to determine whether lowering IS levels or targeting IS-mediated oxidative and inflammatory signaling can attenuate VC and improve cardiovascular outcomes in CKD.

VC is a clinically significant contributor to cardiovascular morbidity and mortality in patients with CKD. Thus, targeting oxidative stress-driven signaling networks rather than individual pathways may more effectively mitigate VC in CKD [43,44]. In particular, modulating ROS generation, ERK activation, and β-catenin signaling may reveal novel approaches for reducing vascular complications associated with uremic toxin accumulation.

Several limitations of this study should be acknowledged. First, although our findings suggest that ROS acts as an upstream mediator linking IS exposure to ERK and Wnt/β-catenin activation, the causal hierarchy among ROS generation, Nrf2 suppression, ERK activation, β-catenin signaling, and RUNX2 induction was not fully established. Although IS reduced the pNrf2/Nrf2 ratio and HO-1 expression, the functional role of Nrf2 suppression was not directly tested using pharmacological Nrf2 activators, such as dimethyl fumarate or sulforaphane, or genetic approaches. In addition, other Nrf2-responsive antioxidant proteins, including NAD(P)H quinone dehydrogenase 1, superoxide dismutase 1, and superoxide dismutase 2, were not evaluated. Therefore, the extent of Nrf2-related antioxidant dysfunction could not be comprehensively characterized. Although NAC attenuated IS-induced ROS accumulation and cellular injury, its effects on ERK activation, Wnt/β-catenin signaling, osteogenic marker expression, and calcium deposition were not evaluated. Future studies incorporating broader assessment of Nrf2-responsive antioxidant targets together with pharmacological or genetic modulation of Nrf2 are needed to clarify the causal hierarchy linking oxidative stress to downstream procalcific signaling. Second, pharmacological inhibitors, including DKK-1 and PD98059, may have off-target effects; therefore, genetic inhibition or overexpression experiments are required to confirm the pathway-specific roles of Wnt/β-catenin and ERK signaling. Third, although MOVAS cells provide a useful in vitro model for studying VSMC calcification, immortalized cell lines may not fully reproduce the phenotype and responses of primary VSMCs or human vascular tissues. Furthermore, although ALP, RUNX2, BMP2, and OCN were evaluated, other relevant markers of osteogenic differentiation and VSMC phenotypic switching, including PiT-1, Osterix, and the contractile marker SM22α, were not assessed. Therefore, the present marker panel supports osteogenic and calcific responses but does not comprehensively characterize the transition from a contractile to an osteogenic phenotype. In addition, an ex vivo aortic culture model was not included. Such a model would preserve vascular tissue architecture and cell–matrix interactions and provide a more physiologically relevant assessment of the direct effects of IS on medial calcification. Future studies incorporating primary VSMCs, broader osteogenic and contractile marker panels, and ex vivo aortic tissue cultures are warranted to validate and extend the present findings. Fourth, the IS concentrations used in vitro may not completely reflect the complex exposure patterns observed in patients with CKD, in whom total and free IS levels, protein binding, duration of exposure, and interactions with other uremic toxins may vary substantially. Fifth, although the nephrectomy-induced CKD mouse model reproduces important features of renal dysfunction and VC, it cannot fully capture the heterogeneity of human CKD, including differences in etiology, comorbidities, mineral metabolism, medication use, dialysis status, and dietary factors. In addition, aortic calcification was assessed using section-based von Kossa staining rather than direct biochemical measurement of tissue calcium or whole-mount Alizarin Red staining of the aortic arch and descending aorta. Although anatomically matched sections with a consistent sectioning plane were used in the revised analysis, histological quantification may still be influenced by tissue orientation, section selection, and regional heterogeneity of calcification. Future studies should incorporate acid-extraction-based calcium quantification and whole-mount staining to provide complementary and more comprehensive assessments of vascular mineral deposition. Finally, this study did not include human clinical samples; therefore, the clinical relevance of the proposed IS–oxidative stress–ERK/Wnt/β-catenin axis requires further validation in patient-based studies. Future longitudinal, interventional, and translational studies are warranted to determine whether reducing IS accumulation or targeting IS-mediated oxidative and inflammatory signaling can attenuate VC in CKD.

## 4. Materials and Methods

### 4.1. Cell Culture

MOVAS cells (CRL-2797; American Type Culture Collection [ATCC], Manassas, VA, USA) were cultured in high-glucose Dulbecco’s Modified Eagle Medium (Life Technologies, Gaithersburg, MD, USA) supplemented with 10% fetal bovine serum (Life Technologies, Gaithersburg, MD, USA) and 0.2 mg/mL G418 (Sigma-Aldrich, St. Louis, MO, USA). Cells were maintained at 37 °C in a humidified incubator with 5% CO_2_ and were subcultured every 2 days in the appropriate medium. All experiments were performed using at least three independent biological replicates.

### 4.2. Calcification Induction and Experimental Treatments

For the time-course calcification experiments shown in Figure 3, MOVAS cells were treated with IS (Sigma-Aldrich, St. Louis, MO, USA) at concentrations of 0, 125, 250, or 500 μM for 7, 14, or 21 days. The culture medium containing the corresponding concentration of IS was replaced every 2 days [45]. For time-course experiments, the first day of exposure to the calcification medium was designated as day 1. No additional calcification-inducing agents, including β-glycerophosphate, inorganic phosphate, calcium chloride, ascorbic acid, or dexamethasone, were added.

For the ROS, cell viability, Western blotting, and OCN ELISA experiments shown in Figure 4 and Figure 5, MOVAS cells were treated with IS at concentrations of 0, 125, 250, or 500 μM. For antioxidant experiments, MOVAS cells were pretreated with 2 mM N-acetyl-L-cysteine (NAC, Sigma-Aldrich, St. Louis, MO, USA) for 1 h before exposure to 500 μM IS. Cells were subsequently cultured in calcification medium containing both IS and NAC for 21 days. For pathway-inhibition experiments, MOVAS cells were exposed to 500 μM IS and subsequently treated with the ERK inhibitor PD98059 (MedChemExpress, Monmouth Junction, NJ, USA) at 25 or 50 μM or recombinant DKK-1 (MedChemExpress, Monmouth Junction, NJ, USA) at 50 or 100 μg/mL. Protein lysates were collected 2 h after inhibitor treatment.

### 4.3. Alizarin Red S Staining

Cells were fixed with 95% ethanol for 30 min at room temperature and stained with 0.1% Alizarin Red S solution (pH 4.3, Abcam, Cambridge, UK) at 37 °C. Calcified nodules were visualized under a light microscope. To quantify calcium deposition, the bound dye was eluted with 10% formic acid, and the absorbance was measured at 420 nm using a microplate reader.

### 4.4. Western Blotting

Total protein was extracted from MOVAS cells using radioimmunoprecipitation assay lysis buffer supplemented with protease and phosphatase inhibitor cocktails. Protein samples were centrifuged at 12,000× *g* for 30 min, and the concentrations were determined using a Bio-Rad protein assay (Bio-Rad Laboratories, Hercules, CA, USA). Equal amounts of protein were separated by sodium dodecyl sulfate–polyacrylamide gel electrophoresis and transferred onto polyvinylidene fluoride membranes. Membranes were blocked with 5% nonfat milk and incubated overnight at 4 °C with primary antibodies against RUNX2 (Abcam, Cambridge, UK), Nrf2, phosphorylated Nrf2, IL-6, BMP2, ALP, and HO-1 (ABclonal, Woburn, MA, USA), β-catenin (Cell Signaling Technology, Danvers, MA, USA), and ERK1/2 and phospho-ERK1/2 (Invitrogen, Carlsbad, CA, USA). After incubation with horseradish peroxidase-conjugated secondary antibodies, signals were detected using enhanced chemiluminescence (GE Healthcare, Chicago, IL, USA) and visualized using an iBright 1500 imaging system (Thermo Fisher Scientific, Waltham, MA, USA). Protein expression was quantified using ImageJ software (version 1.52a; National Institutes of Health, Bethesda, MD, USA) and normalized to β-actin (ABclonal, Woburn, MA, USA).

### 4.5. Reactive Oxygen Species and Live/Dead Cell Analyses

Intracellular ROS levels were measured using 2′,7′-dichlorodihydrofluorescein diacetate (DCFDA; Abcam, Cambridge, UK). Following treatment, cells were incubated with DCFDA and analyzed by flow cytometry. Cell viability was assessed using the Zombie NIR™ Fixable Viability Kit (BioLegend, San Diego, CA, USA). Cells were stained for 30 min in phosphate-buffered saline according to the manufacturer’s instructions and analyzed using a CytoFLEX flow cytometer (Beckman Coulter Life Sciences, Indianapolis, IN, USA).

### 4.6. Osteocalcin Enzyme-Linked Immunosorbent Assay

Intracellular OCN protein levels were quantified using the Mouse OCN SimpleStep ELISA^®^ Kit (Abcam, Cambridge, UK) according to the manufacturer’s instructions. Following the indicated treatments, MOVAS cells were harvested and lysed, and the cell lysates were centrifuged to remove insoluble debris. Total protein concentrations were determined using the Bio-Rad Protein Assay (Bio-Rad Laboratories, Hemel Hempstead, Hertfordshire, UK). Equal amounts of protein were subjected to ELISA analysis, and absorbance was measured at 450 nm using a microplate reader. OCN concentrations were normalized to the total protein content and expressed as ng/mg protein.

### 4.7. Animals

Thirty-two male C57BL/6 mice of the same age (6 weeks old, 20–25 g) were obtained from BioLASCO Taiwan Co., Ltd. (Taipei, Taiwan). All mice were age-matched at the beginning of the experiment and were 6 weeks old at the time of randomization and initial surgery. Mice were randomly assigned to four groups (n = 8 per group): Vehicle, 1/3 nephrectomy (1/3 Nx), 1/2 nephrectomy (1/2 Nx), and 5/6 nephrectomy (5/6 Nx).

All animals were maintained under controlled laboratory conditions with free access to food and water. All animal experimental procedures were approved by the Institutional Animal Care and Use Committee of Hualien Tzu Chi Hospital (approval number: 111-54).

### 4.8. Two-Step Nephrectomy-Induced Chronic Kidney Disease Mouse Model

A two-step nephrectomy-induced CKD mouse model was used in the present study to generate different degrees of renal mass reduction. Briefly, surgical interventions were performed sequentially based on age. At 6 weeks of age, mice underwent right unilateral nephrectomy (for the 1/2 Nx and 5/6 Nx groups) or sham surgery (for the Vehicle and 1/3 Nx groups). At 7 weeks of age, a second surgery was performed on the left kidney, during which a main branch of the left renal artery was ligated in the 1/3 Nx and 5/6 Nx groups to induce partial ischemic infarction. Starting from 8 weeks of age, all mice received daily oral gavage of normal saline. At 16 weeks of age, all animals were euthanized, and blood and tissue samples were collected for further analysis. Based on the extent of renal mass reduction, mice were assigned to the Vehicle, 1/3 Nx, 1/2 Nx, and 5/6 Nx groups. Control animals underwent sham procedures. All surgeries were performed under isoflurane (Attane; Panion & BF Biotech Inc., Taipei, Taiwan) anesthesia [46,47].

### 4.9. Transdermal Glomerular Filtration Rate

At 16 weeks of age, tGFR was assessed using the clearance kinetics of fluorescein isothiocyanate–sinistrin (FITC-sinistrin; Mannheim Pharma & Diagnostics GmbH, Mannheim, Germany), as described previously [46,47]. Hair was removed from the placement site for the tGFR monitor (MediBeacon, St. Louis, MO, USA), which was subsequently attached to the shaved skin. Mice were anesthetized with isoflurane and administered FITC-sinistrin (Mannheim Pharma & Diagnostics, Mannheim, Germany) at a dose of 0.15 mg/g body weight via the retro-orbital venous sinus. tGFR was monitored for 1.5 h and calculated based on the half-life of FITC-sinistrin clearance using MPD Studio software (version RC15, MediBeacon, St. Louis, MO, USA).

### 4.10. Biochemical Analysis

Blood samples were collected from mice at 16 weeks of age and centrifuged at 10,000× *g* for 10 min at 4 °C to obtain the serum. BUN and creatinine serum levels were measured using an automated biochemical analyzer (Spotchem SP-4430, Arkray, Minneapolis, MN, USA) according to the manufacturer’s instructions.

### 4.11. Determination of Indoxyl Sulfate Levels by High-Performance Liquid Chromatography–Mass Spectrometry

Serum IS concentrations were measured using a modified method of high-performance liquid chromatography (HPLC)–tandem mass spectrometry, as described previously [48]. Briefly, 100 µL serum was mixed with 100 µL of 50 mM sodium phosphate dibasic heptahydrate, which was added with d4-IS (Sigma-Aldrich, St. Louis, MO, USA) as the internal standard. Protein extraction was performed using Novum^®^ simplified liquid extraction cartridges (Phenomenex, Torrance, CA, USA), and IS was eluted with 1.5 mL ethyl acetate. The mixture was evaporated under nitrogen to obtain the residue, which was then reconstituted in 100 µL methanol. Chromatographic separation was performed using a Waters^®^ e2695 HPLC system coupled with an ACQUITY QDa^®^ single-quadrupole mass detector (Waters Corporation, Milford, MA, USA) and a Phenomenex Luna^®^ C18(2) column (5 µm, 250 × 4.6 mm, 100 Å) maintained at 40 °C. The flow rate was 0.8 mL/min, and the injection volume was 30 µL. The mobile phases consisted of water and methanol, each containing 0.1% formic acid. The gradient was programmed from 5% to 70% methanol over 14 min, held for 2 min, and then returned to 50% over 2 min. IS and d4-IS were detected in negative electrospray ionization mode, with selected ion recording at *m*/*z* 211.9 for IS and *m*/*z* 216.0 for d4-IS. Serum IS concentrations were quantified using calibration curves generated from d4-IS (Sigma-Aldrich, St. Louis, MO, USA) solutions.

### 4.12. Aortic Tissue Preparation and Von Kossa Staining

Mice were euthanized by carbon dioxide inhalation. Carbon dioxide was introduced into an 8.5 L chamber at a flow rate of 3.4 L/min, corresponding to 40% of the chamber volume per minute, to minimize respiratory distress and facilitate rapid loss of consciousness, in accordance with the 2020 American Veterinary Medical Association Guidelines for the Euthanasia of Animals. Segments of the descending aorta were collected from the same predefined anatomical region in all mice, fixed in 4% buffered formaldehyde, embedded in paraffin, and subjected to von Kossa staining (Abcam, Cambridge, UK). After deparaffinization and rehydration, tissue sections were incubated in 5% silver nitrate solution for 60 min under a 100 W incandescent lamp. The sections were then treated with 5% sodium thiosulfate for 2 min, counterstained with nuclear fast red for 5 min, and rinsed with distilled water before and after each incubation step. Finally, the slides were dehydrated, mounted, and examined by light microscopy. Calcium deposition was quantified as the integrated optical density per unit area (IOD/μm^2^) of von Kossa-positive staining using Image-Pro Plus software, version 6.0 (Media Cybernetics, Rockville, MD, USA) [46,47].

### 4.13. Statistical Analysis

Data are expressed as mean ± standard deviation for in vitro experiments or mean ± standard error of the mean for in vivo experiments. Comparisons among multiple groups were performed using one-way analysis of variance, followed by Tukey’s multiple-comparisons test or Dunnett’s multiple-comparisons test. Data were analyzed using two-way analysis of variance, with IS concentration and treatment duration as the two factors, followed by Šídák’s multiple-comparisons test for prespecified comparisons within each time point. A *p*-value <0.05 was considered statistically significant. Statistical analyses were conducted using GraphPad Prism version 9.0 (GraphPad Software, Boston, MA, USA).

## 5. Conclusions

In conclusion, this study demonstrated that IS promotes osteogenic and calcific responses in VSMCs, as reflected by increased calcium deposition and elevated ALP, RUNX2, BMP2, and OCN levels, accompanied by oxidative stress-related activation of ERK and Wnt/β-catenin signaling and suppression of Nrf2-related antioxidant defenses.

## Data Availability

The data generated and analyzed in this study are available upon reasonable request from the corresponding author.

## Abbreviations

The following abbreviations are used in this manuscript:

AhR	Aryl hydrocarbon receptor
ALP	Alkaline phosphatase
ARRIVE	Animal Research: Reporting of In Vivo Experiments
ATCC	American Type Culture Collection
BMP2	Bone morphogenetic protein 2
BUN	Blood urea nitrogen
CKD	Chronic kidney disease
DCFDA	2′,7′-dichlorodihydrofluorescein diacetate
DKK-1	Dickkopf-1
ELISA	Enzyme-linked immunosorbent assay
ERK	Extracellular-signal-regulated kinase
FITC	Fluorescein isothiocyanate
HO-1	Heme oxygenase-1
HPLC	High-performance liquid chromatography
IL-6	Interleukin-6
IOD	Integrated optical density
IS	Indoxyl sulfate
MAPK	Mitogen-activated protein kinase
MOVAS	Mouse aortic vascular smooth muscle
NAC	N-acetyl-L-cysteine
Nrf2	Nuclear factor erythroid 2-related factor 2
Nx	Nephrectomy
OCN	Osteocalcin
ROS	Reactive oxygen species
RUNX2	Runt-related transcription factor 2
tGFR	Transdermal glomerular filtration rate measurement
VC	Vascular calcification
VSMCs	Vascular smooth muscle cells
